# Communications Principles for Inviting Inquiry and Exploration Through Science and Data Visualization

**DOI:** 10.1093/icb/icy105

**Published:** 2018-10-06

**Authors:** Eric Rodenbeck

**Affiliations:** Stamen Design, 2017 Mission Street No. 300, San Francisco, CA 94110, USA

## Abstract

Science, in the popular imagination, is about finding answers to questions. Scientists make discoveries, develop theories, and deliver those discoveries and theories to audiences with an interest in the truth as backed up by science. Well-designed data visualization (dataviz), by contrast, can generate and address not only new questions but new kinds of questions. It has the particular quality of allowing its viewers, users, and makers the ability to generate new inquiries, and to put them in a better place to answer them. Dataviz offers esthetic and interactive platforms for discussion and inquiry that can help scientists to both do their work and better communicate their work to broader audiences. Here I will illustrate and examine case studies from multiple points along the rich and varied possibility space that opens up when science and dataviz work together. I will also introduce three communication principles that I have learned from my involvement with hundreds of dataviz projects over the years. Well-designed dataviz can help scientists and those involved with science find ways to navigate the multiple competing interests and priorities inherent in both communication to non-scientists and exploratory data-rich interfaces.

## Introduction

**Fig. 1:**
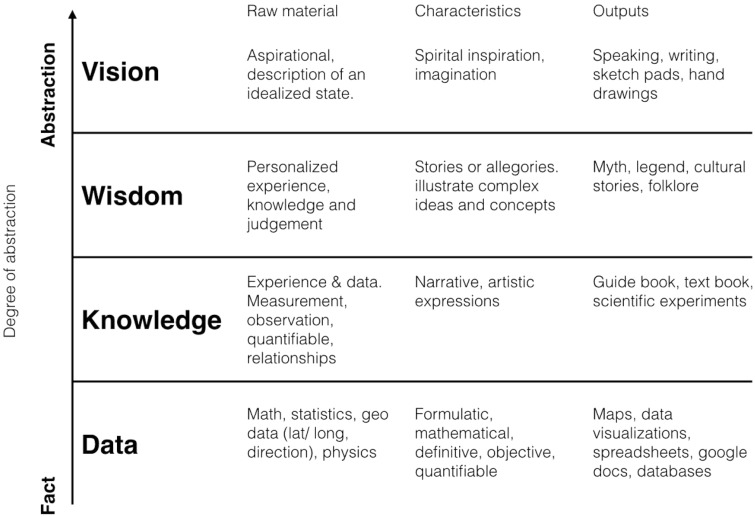
Matrix of data visualization abstraction (Stamen Design 2018).

The focus of dataviz can be understood to exist along a spectrum of abstraction, from facts at the most concrete end, to wisdom, knowledge, and even vision as the most aspirational place for dataviz to work (Fig. 1). Each of these kinds of work requires a different approach, and each uses a different kind of raw material and has unique characteristics and outputs.

Much of what is widely considered dataviz by practitioners from Edward Tufte to Cole Nussbaumer Knaflic focuses exclusively on the Data row in Fig. 1 of this paper. Through our client-facing and research practice at Stamen, we engage in multiple kinds of dataviz approaches across this spectrum. Much of this work is done for and with scientists across a broad range of fields, from metagenomics to the study of human emotions. Thinking of dataviz as more than the communication of facts in the clearest way possible to everyone who looks at it is crucial to unlocking the full communication potential of the medium. There is more to dataviz than communicating simply.

One example of this is the visualization of complex metagenomic data that the scientists at the Banfield Laboratory at the University of California use to analyze new landscapes of genetic diversity. Their work is difficult to explain to lay audiences, due to its complexity. There is a significant gap between how most people think about metagenomic sequencing and what the science involves. Banfield scientists use data visualizations that Stamen built for them for “hypothesis generation and experimentation” (Stamen Design 2016c). This is data visualization for highly skilled and experienced scientists. It requires their personalized experience, knowledge, and judgment in aiding their work, and belongs more properly in the Wisdom row of Fig. 1 than in the Data row. The interfaces are very difficult for lay people to understand, but serve a crucial role in helping the scientists to look at “at all the recovered organisms and all metabolic reactions of these organisms simultaneously” (Stamen Design 2016c). Meeting your audience where they are, whether at a very low or very high degree of scientific literacy or knowledge abstraction, is key to designing interfaces and visualizations that will help achieve your communications goals. What works in one row will likely not work as well in another.

## The intersection of science, data, and the Internet

We are living through an astonishing transformation in the amount and availability of data to people everywhere, both inside and outside of academic institutions. This transition is changing the way that science is done and communicated.

As an example, consider the 2008 paper “Magnetic alignment in grazing and resting cattle and deer.” Researchers analyzed the position of thousands of grazing animals found on Google Earth and found that deer and cattle tend to align their bodies in a north–south direction: “Amazingly, this ubiquitous phenomenon does not seem to have been noticed by herdsmen, ranchers, or hunters” (Begall et al. 2008).

Humans have been looking at deer and cattle for a long time, and yet apparently never noticed the direction in which they tend to stand. The emergence of fast, free, and easy access to an accurate and often-updated library of satellite imagery enables observers to ask different kinds of questions than have been previously addressable. While scientists don’t yet understand the proximate mechanisms behind this behavior, a statistically sufficient sample dataset now exists for other researchers to build on this work. A 2013 paper, also using Google Earth imagery as the source material, found that “mutual distances between individual animals within herds (herd density) affect their N–S preference” (Slaby et al. 2013).

In both these projects, the amount of free and easily available data was the key factor in allowing these insights. There are many other areas where the amount of data available has grown dramatically in recent years, and the field of data visualization is emerging as a set of practices around doing, communicating about, and otherwise dealing with this rapidly changing landscape and possibility space.

This paper presents three communication principles and projects drawn from Stamen’s client services practice visualizing scientific data. These principles can be useful for scientists and the broader public with science through data visualization.

### Principle 1: Public conversations about science are never just about the truth. It’s wise to plan for this, and not shrink from it

**Fig. 2:**
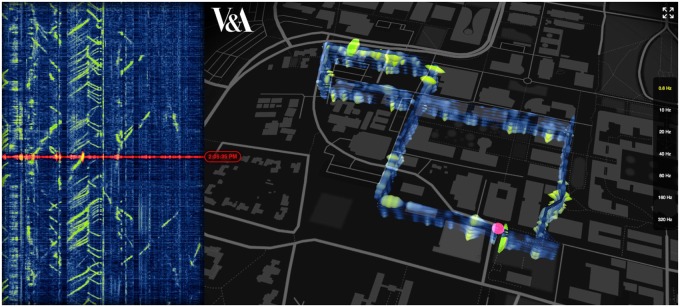
Big Glass Mic. Stamen design. Commissioned by the Victoria and Albert Museum (2017). A provocation that uses material from the “Data” row in Fig. 1 as a source for a project in the Wisdom row, in order to evoke a sense of wonder and mythic imagination applied to unseen infrastructure.

Big Glass Microphone (Fig. 2) is a commission Stamen received from the Victoria and Albert Museum in London (Stamen Design 2017). Relying on the work of Biondo Bondi and Eileen Martin at Stanford, the Stanford Exploration Project and the School of Earth, Energy & Environmental Sciences, the project uses data from a 5 km long fiber optic cable buried about a meter under the ground at Stanford University. Light shines through the cable, which responds to vibrations in the environment by changing its shape very slightly. The Stanford team has shown that it’s possible to convert the vibrations of the perturbed optical fiber strands into information about the direction and magnitude of seismic events.

We received a 9-min long sample of data from the Stanford team. We visualized the data in an interactive, dynamic interface overlaid on a map of the portion of the Stanford Campus under which the cable is buried. The data are split into different frequency bands, ranging from 0.6 to 320 Hz. Each of these bands can be turned on and off, and perturbations in the light waves are clearly visible as bright spots along the length of the cable.

For example, gasoline-powered cars driving on the road above the cable are visible across a range of the frequency bands. Electric cars, which are mostly silent to the naked ear, leave a noticeably different signature. Other perturbation sources, like bicycles, pedestrians, seismic activity, construction equipment, and air conditioners, have different profiles, moving at different speeds than cars. Some perturbations don’t move at all in space, but show significant variability of vibration across multiple frequency bands. By comparing the location of this pattern to the geographical map, we determined that one of these stationary but highly varied objects was a fountain, burbling outside the Earth Sciences Building.

If a fiber optic cable buried under the ground can be used to detect fountains and cars, what else can be used as a sensor? What is equivalent to the Google Earth example above, when this kind of information is as ubiquitous as satellite imagery? What kinds of problems can we use for these sensing apparatuses to investigate? What kinds of new questions could they enable?

Big Glass Microphone is a datavis provocation, done under the guise of art and design, designed to provoke more questions than it answers. It received significant press attention, some of which presented the science behind the project in ways that were not quite accurate and which certainly would not withstand peer review. In particular, some of the articles suggested that the cable could pick up and distinguish human speech, which the researchers have emphasized is not the case. Some of these articles introduced the idea that scientific instrumentation can be used in ways that it was not originally intended for, that this can result in some interesting new kinds of observations: “The fiber optic loop under Stanford’s campus was originally installed last August for seismic research, but the Stanford team, with the help of OptaSense, decided to turn the noise into signals” (Saplakoglu 2017).

The project also opened up the idea for people that this data could be everywhere, and “At a larger scale, imagine how valuable this type of data would be for an engineer corralling traffic, or a mobility service sniffing out customers” (Bliss 2017). From Stamen’s perspective, this is a part of the process of engaging in public conversation: not everyone gets all the facts right, some articles are outright wrong, and information can be taken out of context. Articles written in the lay press have a significantly lower threshold for veracity than those accepted in peer-reviewed journals, by design. Communication with these outlets require different strategies than those commonly deployed by the scientific press, and different strategies are needed to have conversations with those outside the academy.

A key part of this strategy is acknowledging that a natural part of a big public conversation is that not everyone who writes about a project will get every detail exactly correct. It’s not important for every journalist to understand every factual argument that a scientist makes in order to start a useful conversation about science. “You can’t turn a no to a yes without a maybe in between,” Frank Underwood says in House of Cards (Foley 2015). One of the main things I hope to help scientists understand is that they can learn from Frank Underwood in House of Cards when it comes to communicating about their work.

The press will always get something “wrong,” from a scientific perspective. The magnetic alignment projects at the beginning of this article have received widespread press coverage. The 2013 paper occasioned an article (Bates 2013) about both of the projects in Wired magazine titled “Cow Compass Points the Way North.” It’s a goofy, catchy title about a complex topic involving real science and sophisticated research—which is exactly the point. It’s not entirely accurate. But it’s in WIRED. The public is engaged with the work. From a communications perspective, that’s more important than whether they get every single detail about the project correct the first time. This work, though it directly engages with data from a fiber optic cable and might seem to belong in the Data row of Fig. 1, is more useful to think about as a deliberate treatment of a Data project and as a Wisdom project from a communications perspective. It is intended to evoke a sense of wonder and mythic imagination applied to unseen infrastructure.

### Principle 2: Data visualization can invite more questions than it answers

**Fig. 3:**
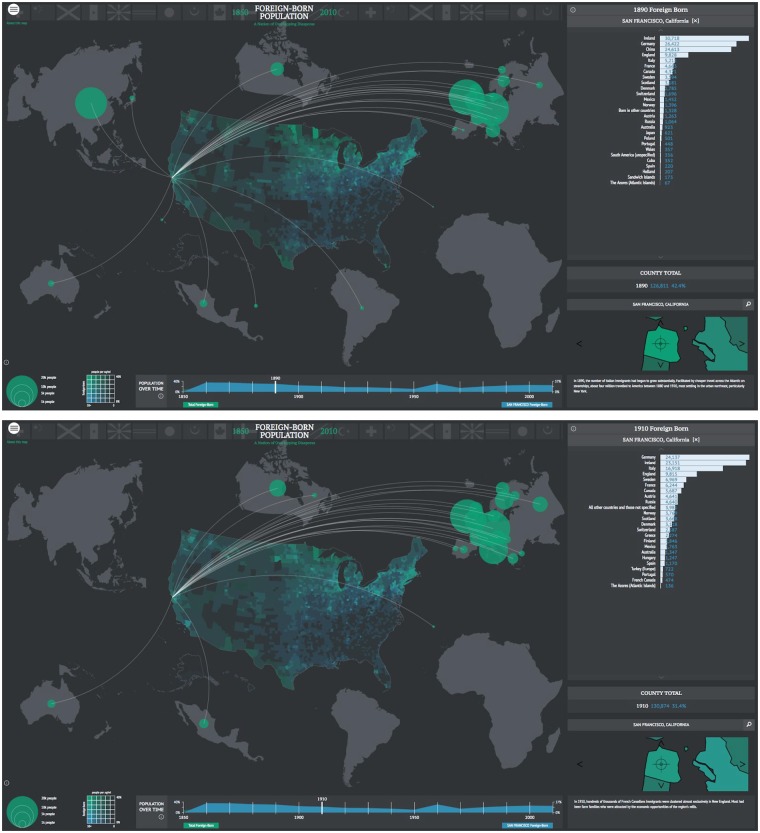
American Panorama: foreign born. Census figures for foreign born population of San Francisco in 1890 (above) and 1910 (below). Stamen Design. Commissioned by the DSL at the University of Richmond (2016). Built by and for expert historians, this project and the principle that informs it also belong more in the Knowledge row of Fig. 1 than in the Facts row.

American Panorama (Fig. 3) is an interactive atlas of American History, designed and built by Stamen (Stamen Design 2016a), and commissioned by the University of Richmond’s Digital Scholarship Lab (DSL). The project uses maps and data visualizations to enable discussion of, and citation of, spatial relationships in different historical contexts. Built by and for expert historians, this project and the principle that informs it also belong more in the Knowledge row of Fig. 1 than in the Facts row.

One of the maps, Foreign Born, displays the number of Americans counted in each census that were born outside the United States, subdivided into counties. Viewers can see that in 1870 in San Francisco close to half the population was born outside the country, with the largest numbers of this group coming from Ireland, Germany, and China. These numbers and proportions remain relatively stable through the 1900 census for San Francisco. Note that each link takes you to a different state of the map, an important detail when citing these maps. In 1910, Chinese immigrants, who were for several decades one of the top three foreign born groups in San Francisco, suddenly disappear from the map. The same thing occurs in 1920, 1930, and 1940. There appear to be no Chinese at all. We see them reappear in much-reduced numbers in the 1950 census. The 1960 census groups all Americans of Asian descent into Asia (unspecified). Chinese are listed as the biggest group of foreign born Americans in San Francisco in 1970, where they remain until the 2010 census, which is the most recent as of this writing.

We know that Chinatown in San Francisco was an active site of Chinese activity during these years. It made no sense that the data showed zero Chinese born Americans during this time. We therefore thought there was a bug in our code. Perhaps we’d spelled something wrong in the latest compile. But when we looked at the code, everything checked out. We went in and looked at the data: the row for China was empty in the data we’d received from the DSL. We finally asked our clients at the University for clarification. The Chinese Exclusion Act of 1882 (Dunigan 2017) not only severely restricted immigration from countries like China. The Act also forbade non-white foreign born people from being counted in the census. They therefore are literally off the map.

The scholars at the DSL asked us to remain open to the possibilities of letting project viewers ask questions of the material. We decided together to leave the gap in the data in the project, as a way to invite inquiry into the material. This was deemed more aligned with the project’s goals as a tool for researchers than to explain every aspect of what the data showed. Sometimes (as in this instance) blank spaces on maps are as important as the parts that are filled in. Sometimes noise is as interesting as signal.

### Principle 3: Data visualization communication is never context-free. There’s no neutral or correct way to do this work

**Fig. 4:**
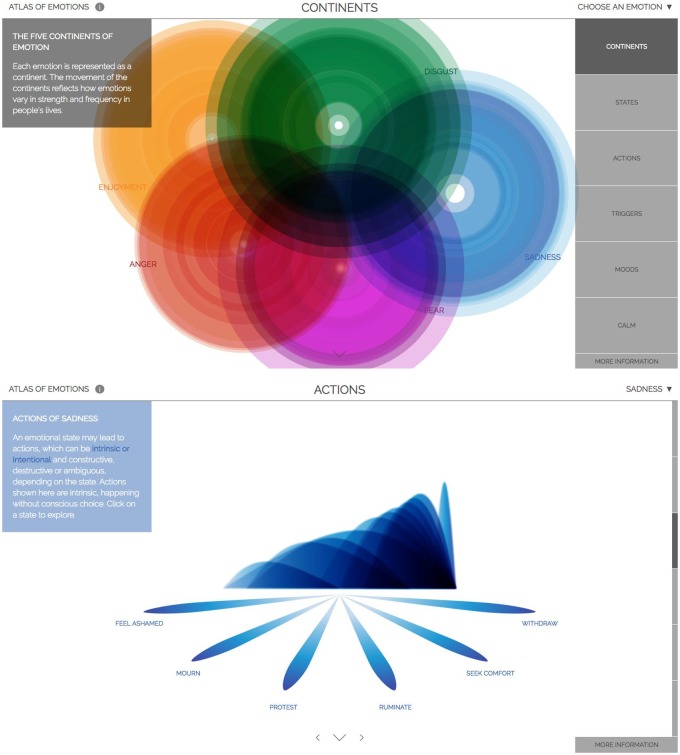
The atlas of emotions. Each emotion presented as an independent continent (above) and the states of Enjoyment displayed along with their attendand Actions (below). Stamen design. Commissioned by the Lama and Ekman (2016). This work belongs in the Vision row of Fig. 1, though the principle that informs it can be equally well-applied to any of the rows in that figure.

We worked with behavioral scientists Paul and Eve Ekman on The Atlas of Emotions (Fig. 4), commissioned by the Dalai Lama. His Holiness and Paul have written several books together about emotions, bringing their differing world views to bear on the subject of emotions to the benefit of both. The Dalai Lama, for example, learned about the concept of mood, an emotional state that causes people to interpret various events through the lens of an emotion that may not be the appropriate one for the task at hand. This was a new notion for him because, in Tibetan Buddhism, there is no concept for a bad mood (Lama and Ekman 2009). And Paul learned about the Tibetan concept of attachment as a kind of fulcrum point between emotional aversion and emotional attraction. The Dalai Lama knows that, while he can speak to a certain kind of audience whose disposition might lead them to listen to what he has to say, others, perhaps those more scientifically minded, might be suspicious of the message a Buddhist monk might bring. The Dalai Lama therefore asked Paul to design for him an atlas of what science knows about how emotions work to address this need. Paul asked us to help him design and build it. This work belongs in the Vision row of Fig. 1, though the principle that informs it can be equally well-applied to any of the rows in that figure.

Paul’s response was to design a survey to uncover the consensus among scientists who study emotion, and what they disagree about. According to the survey, scientists agree that all humans share five emotions: anger, fear, sadness, disgust, and enjoyment. We then worked with Paul and Eve to design a visual representation of what these data showed, drawing on these scientists’ understanding of the structure and nature of human emotions.

The project identifies emotional states within each primary emotion, organized by their felt intensity. Among the multiple states of anger, for example, annoyance is felt only at the lower levels of intensity. Exasperation and bitterness, two other states of anger, bridge very low levels of intensity and very high levels of intensity. Fury, the most intense state of anger, is only ever felt at the highest levels. You cannot be slightly furious.

Paul had never looked at his work this way before, which was a surprise to both him and us. This renowned scientist, whose work laid the foundation for the modern scientific understanding of emotions, had never taken the time to count or map the relative intensities of the different emotional states he’d been studying for 60 years. Far from indicating a gap in Paul’s work, what we feel that this demonstrates is an powerful example of an opportunity for designers and scientists to work together to help bring a new level of visual thinking and accessibility to the vital work of science. Paul later commented that this collaboration was

… wasn’t just about discovering things I didn’t know about my own research … I also learned things that I didn’t think it was possible to know about my work. (Stamen Design 2016b)

The project was designed for an English-speaking, Western audience, as the Dalai Lama had asked us to do. This included color choices, which happened to be the same colors chosen for the five emotions that live in the character of Riley’s head in the recent Pixar movie Inside Out. We chose green for disgust, blue for sadness, orange for enjoyment, purple for fear, and red for anger.

When talking about the project, I often used the example of how red is a symbol of good luck in China to illustrate what we assumed was large variance of opinion on color-emotional correspondence in other parts of the world. Surely the Chinese would have a different color for anger. How could an emotion generally associated with negativity also be associated with good luck? To my surprise, when I finally had the chance this year to ask a group of Chinese speakers what color they associated with anger, they all said the same thing: red. They also told me that they associate the color blue with sadness, but that this was likely only true in China since the introduction of Elvis Presley’s music.

## Conclusion

In this new world of data ubiquity, scientific and dataviz communication is like any other kind of communication. There are many opportunities available and examples to choose from as you decide how to work with data and communicate with it, both to your scientific peers and to the public. Evaluating dataviz science communication based only on whether the public immediately understands all the important aspects to the science can serve as a barrier to more widespread understanding of the work. The continuum of types of dataviz (Fig. 1) can serve as a useful framing device for making decisions about how to communicate in different ways to different kinds of audiences. It’s important to note that these principles and data types are by no means definitive. As has been discussed in relation to Big Glass Mic, useful results can come from applying the lessons from one technique to a project that would seem to better fit in another. Nevertheless, by considering these principles and actively employing them when communicating about their work to lay audiences, scientists can have a greater impact on the world than by adhering to the same strict principles of scientific accuracy that we expect them to deploy in their research.
